# Adenovirus Serotype 5 Vectors with Tat-PTD Modified Hexon and Serotype 35 Fiber Show Greatly Enhanced Transduction Capacity of Primary Cell Cultures

**DOI:** 10.1371/journal.pone.0054952

**Published:** 2013-01-25

**Authors:** Di Yu, Chuan Jin, Mohanraj Ramachandran, Jing Xu, Berith Nilsson, Olle Korsgren, Katarina Le Blanc, Lene Uhrbom, Karin Forsberg-Nilsson, Bengt Westermark, Rachel Adamson, Norman Maitland, Xiaolong Fan, Magnus Essand

**Affiliations:** 1 Department of Immunology, Genetics and Pathology, Science for Life Laboratory, Uppsala University, Uppsala, Sweden; 2 Division of Clinical Immunology, Karolinska University Hospital, Huddinge, Sweden; 3 Department of Biology, YCR Cancer Research Unit, University of York, Heslington, United Kingdom; 4 Rausing Laboratory, Lund University, Lund, Sweden; 5 Beijing Key Laboratory of Gene Resource and Molecular Development, College of Life Sciences, Beijing Normal University, Beijing, China; University of Chicago, United States of America

## Abstract

Recombinant adenovirus serotype 5 (Ad5) vectors represent one of the most efficient gene delivery vectors in life sciences. However, Ad5 is dependent on expression of the coxsackievirus-adenovirus-receptor (CAR) on the surface of target cell for efficient transduction, which limits it’s utility for certain cell types. Herein we present a new vector, Ad5PTDf35, which is an Ad5 vector having serotype 35 fiber-specificity and Tat-PTD hexon-modification. This vector shows dramatically increased transduction capacity of primary human cell cultures including T cells, monocytes, macrophages, dendritic cells, pancreatic islets and exocrine cells, mesenchymal stem cells and tumor initiating cells. Biodistribution in mice following systemic administration (tail-vein injection) show significantly reduced uptake in the liver and spleen of Ad5PTDf35 compared to unmodified Ad5. Therefore, replication-competent viruses with these modifications may be further developed as oncolytic agents for cancer therapy. User-friendly backbone plasmids containing these modifications were developed for compatibility to the AdEasy-system to facilitate the development of surface-modified adenoviruses for gene delivery to difficult-to-transduce cells in basic, pre-clinical and clinical research.

## Introduction

Adenovirus serotype 5 (Ad5) is promising as a genetic vaccine vector and a gene delivery vehicle due to its non-integrating episomal gene expression and ability to transduce both dividing and non-dividing cells. Furthermore, replicating adenoviruses are being developed as oncolytic agents for cancer gene therapy. However, Ad5 is dependent on expression of the coxsackievirus-adenovirus-receptor (CAR) on the surface of target cell for efficient transduction. Since CAR is expressed at low levels on some human primary cell types, especially cells of hematopoietic origin, and CAR expression is frequently downregulated on tumor cells, Ad5 transduction is often not efficient. Extensive efforts have been devoted to improve the therapeutic efficacy of adenovirus by modifying adenoviral surface proteins, which include fiber pseudotyping and/or modification [1], [2], [3], [4], pIX modification [5], [6] hexon modification [7], [8] and also chemically mediated surface modifications [9]. We have previously reported that modified Ad5 containing the protein transduction domain (PTD) from the HIV-1 Tat protein (Tat-PTD) inserted into the hexon hyper variable region 5 (HVR5) of the virus capsid, have significantly higher transduction capacity than wild-type Ad5 and show increased oncolytic efficacy on many tumor cell types including neuroendocrine tumors and neuroblastoma [10]. Several groups have reported that CD46, the primary receptor for adenovirus serotype 35 (Ad35) is expressed on most human cells throughout the body and frequently upregulated on tumor cells [11], [12], [13]. Herein, we show that by switching the adenovirus fiber from serotype 5 to serotype 35 on the Tat-PTD-modified vector to generate a new vector, Ad5PTDf35, the transduction efficiency increases in a wide spectrum of human primary cell types.

## Materials and Methods

### Ethics Statement

The Swedish Work Environment Authority has approved the work with genetic modification of the infectious capacity of human adenovirus serotype 5 (ID numbers 202100-2932 v66a13 (laboratory) and v67a9 (mice)). The studies were performed in accordance with national rules and regulations and international guidelines. All experiments regarding modified adenoviruses were conducted under Biosafety level 2. The Local Ethics Boards in Uppsala and Stockholm has approved the use of the human samples included in the study. The Uppsala Animal Ethics Committee has approved the animal studies (ID number C319/9).

### Construction and Production of Surface-modified Adenoviruses

**Figure 1:**
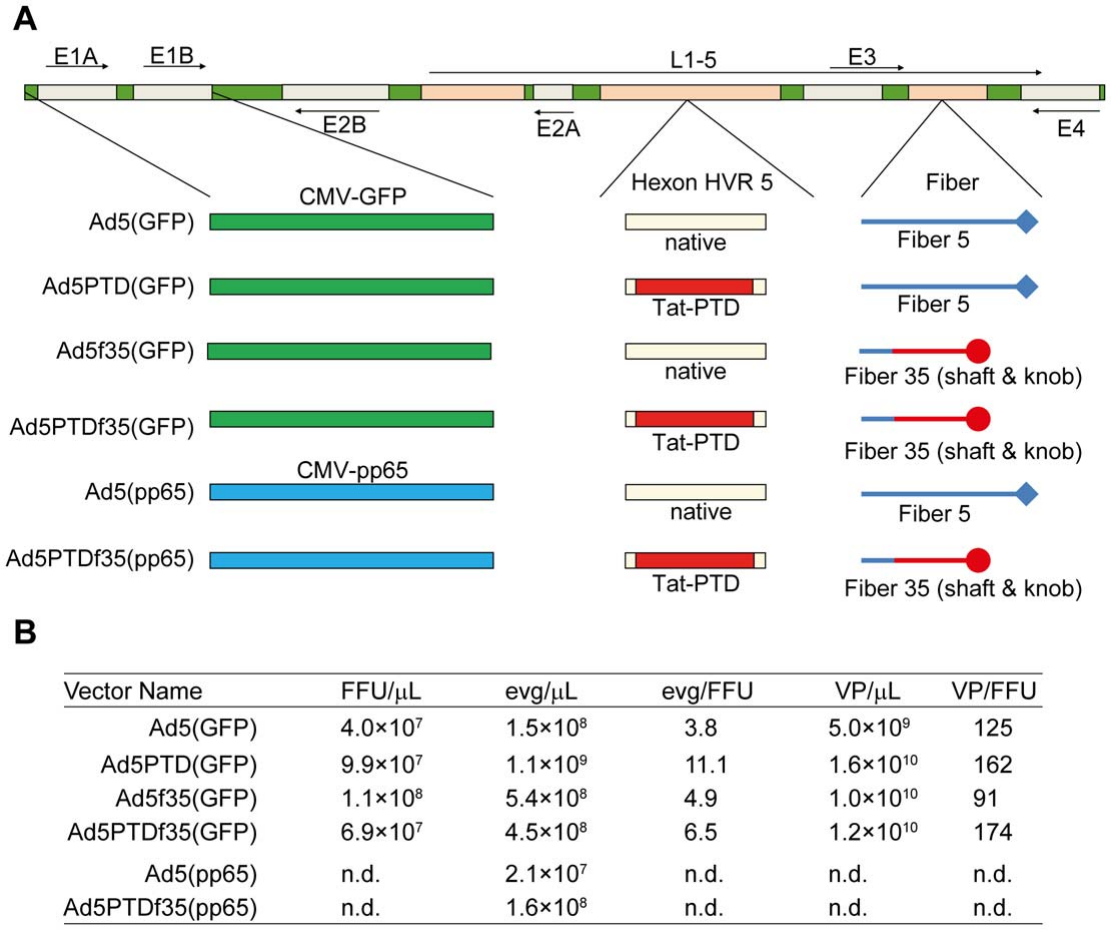
Viral vectors used in this study (A) and their titers (B). (A) A schematic representation of the engineered viruses used in this study. All viruses are based on E1-delated human Ad5. The transgenes (GFP or pp65) are under control of the human cytomegalovirus (CMV) immediate early promoter. The sequence encoding Tat-PTD was inserted into the hexon HVR5 region. The gene encoding the adenovirus fiber shaft and knob was either kept from Ad5 or replaced with the Ad35 counterparts. (B) Titers of the viruses used in this study established by three methods of determination: encapsidated virus genomes (evg) based on quantitative PCR, fluorescent forming units (FFU) based on infection of 911 cells and virus particles (vp) based on optical density measurements.

Recombinant adenoviruses were generated by the means of λ-phage-mediated recombineering in E. *coli* strain SW102 as described previously [10], [14]. The Ad5 fiber shaft and knob regions of Ad5PTD(GFP) were replaced by the serotype 35 counterparts to construct the double-modified Ad5PTDf35(GFP). A double-modified adenoviral vector encoding the cytomegalovirus (CMV) pp65 transgene, Ad5PTDf35(pp65), was also produced. The Ad5(GFP), Ad5PTD(GFP) and Ad5f35(GFP) and Ad5(pp65) vectors have been described previously [10], [15], [16]. Figure 1A shows an illustration of all viral vectors used in this study.

Furthermore, the Tat-PTD sequence was introduced in pAdEasy-1 [17], pAdEasy-1/F35 [15], pAdEasy-1.E3 [18] and pAdEasy-1.F35.E3 (developed in this study, with intact E3 and fiber shaft/knob from serotype 35), in order to produce backbone plasmids for straightforward construction of recombinant adenoviruses using the user-friendly AdEasy system [17], [19]. These plasmids are presented in Table 1. Viruses were produced in 911 cells (Crucell, Leiden, The Netherlands) followed by CsCl gradient purification [20]. Since the virus capsid was modified, the infectious virus titer will depend on the cell line used for titration. Therefore, the viral vectors were titrated as encapsidated viral genome (evg) copy number per µl using quantitative PCR with specific primer detecting the Ad5 E4 region as previously described [10]. Virus titers were also determined by an FFU assay [10] and by viral particle measurements [21] for comparison and to demonstrate the stability of the modified viruses during production (Figure 1B). Each virus was produced once and titrated in parallel.

**Table 1:** pAdEasy backbone vectors for production of recombinant adenovirus.

Vector^1^ name	Hexon	E3^1^	Fiber	Description
pAd5(ΔE3)	Wild-type	Deleted	5	Commercially available as pAdEasy-1
pAd5	Wild-type	Intact	5	pAdEasy-1 with intact E3, for oncolytic virus construction
pAd5PTD(ΔE3)	Tat-PTD^2^	Deleted	5	Targets CAR, increased transduction, for vector construction
pAd5PTD	Tat-PTD^2^	Intact	5	Targets CAR, increased transduction, for oncolytic viruses
pAd5f35(ΔE3)	Wild-type	Deleted	35^3^	pAdEasy-1 with fiber 35, originally named pAdEasy-1/F35
pAd5f35	Wild-type	Intact	35^3^	Targets CD46, for oncolytic viruses
pAd5PTDf35(ΔE3)	Tat-PTD^2^	Deleted	35^3^	Targets CD46, increased transduction, for vector construction
pAd5PTDf35	Tat-PTD^2^	Intact	35^3^	Targets CD46, increased transduction, for oncolytic viruses

1 The backbone plasmids without the adenoviral E3 region are intended for production of vectors for genetic vaccine/gene delivery, while the E3-containing plasmids are more suitable for oncolytic virus development.

2 A Tat-PTD sequence was inserted in hypervariable region 5 of the adenovirus hexon.

3 The fiber (shaft and knob) was replaced with the corresponding sequences from adenovirus serotype 35.

### Primary Cell Isolation and Culture

Peripheral blood mononuclear cells (PBMCs) were isolated from buffy coats from healthy blood donors by Ficoll-Paque (GE Healthcare, Uppsala, Sweden) density gradient centrifugation. They were cultured in RPMI-1640 medium (Invitrogen, Carlsbad, CA) supplied with 10% fetal bovine serum (FBS, Invitrogen). T cells were purified using anti-CD3 magnetic beads (Miltenyi Biotec, Bergisch Gladbach, Germany) according to the manufacturer’s instructions. T cells were activated for 72 hours in culture medium supplemented with OKT-3 (100 ng/ml, BioLegend, San Diego, CA) and interleukin (IL)-2 (50 IU/ml, Proleukin, Novartis, Basel, Switzerland). Monocytes were isolated from PBMCs by plastic adhesion for 90 min [22]. Immature dendritic cells (DCs) were obtained by differentiating monocytes in culture medium supplemented with GM-CSF (50 ng/mL, Gentaur, Brussels, Belgium) and IL-4 (25 ng/mL, Gentaur) for 7 days with fresh medium every second day. Macrophages were obtained by differentiating monocytes in culture medium supplemented with M-CSF (10 ng/mL, R&D Systems, Minneapolis, MN) for 6 days with fresh medium every second day. StemPro Accutase (Invitrogen) was used for cell dissociation from cell clumps or from plastic culture flasks or dishes. Pancreatic exocrine tissue and islets were isolated according to a method described previously [23] and cultured as described previously [24]. Mesenchymal stem cells (MSCs) were isolated from bone marrow as described previously [25] and maintained in DMEM/Glutamax-I (Invitrogen) low glucose medium with 10% FBS (PAA Laboratories, Pasching, Austria). Benign prostatic hyperplasia (BPH) cells and primary prostate cancer cells (Gleason 8 and 9), were isolated from patient samples and cultured as described previously [26]. Human primary high-grade glioma (glioblastoma grade IV) cell cultures (U3013MG and U3054MG where U stands for Uppsala and MG stands for malignant glioblastoma) were isolated from 2 patients and maintained in well-defined serum free medium as described [27].

### Transduction Efficiency Assay

**Figure 2:**
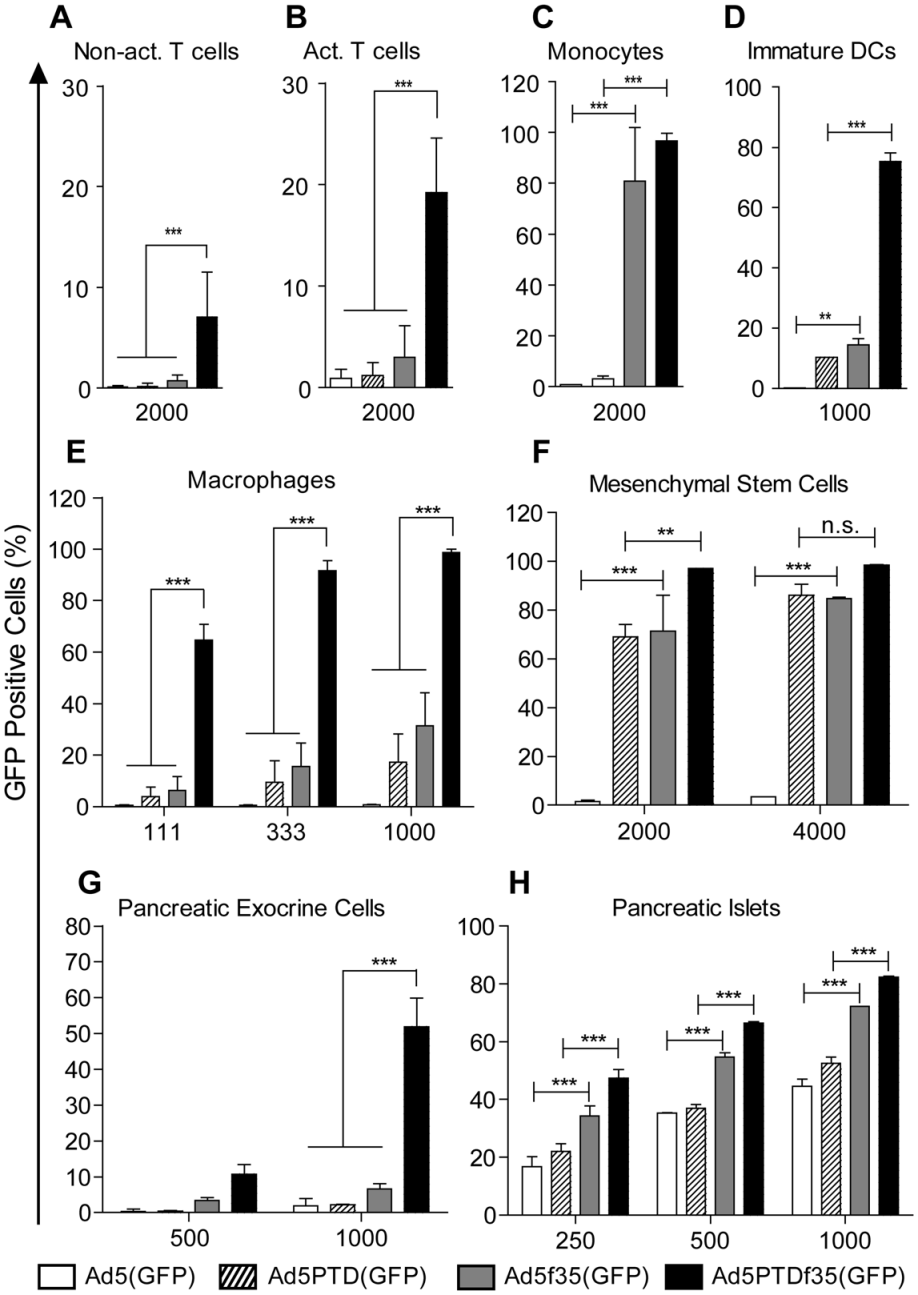
Transduction efficiency of Ad5(GFP), Ad5PTD(GFP), Ad5f35(GFP) and Ad5PTDf35(GFP) in human primary cell cultures. Primary human cells were isolated as described in the materials and methods section and transduced with the four GFP-encoding adenoviral vectors using fixed evg/cell and fixed total volumes. GFP expression was then analyzed by flow cytometry and transduction level is presented as the percentage of GFP-positive cells. The numbers under the x-axes indicate the evg/cell used for transduction. Experiments were repeated on cell cultures from 4–8 different donors except for MSCs, which was repeated 3 times. Non-act.: non-activated. Act.: activated. DCs: dendritic cells. Error bar represents standard deviation. Non-parametric student *t*-test was used for comparison between different groups. *:p<0.05, **:p<0.01, ***:p<0.001.

**Figure 3:**
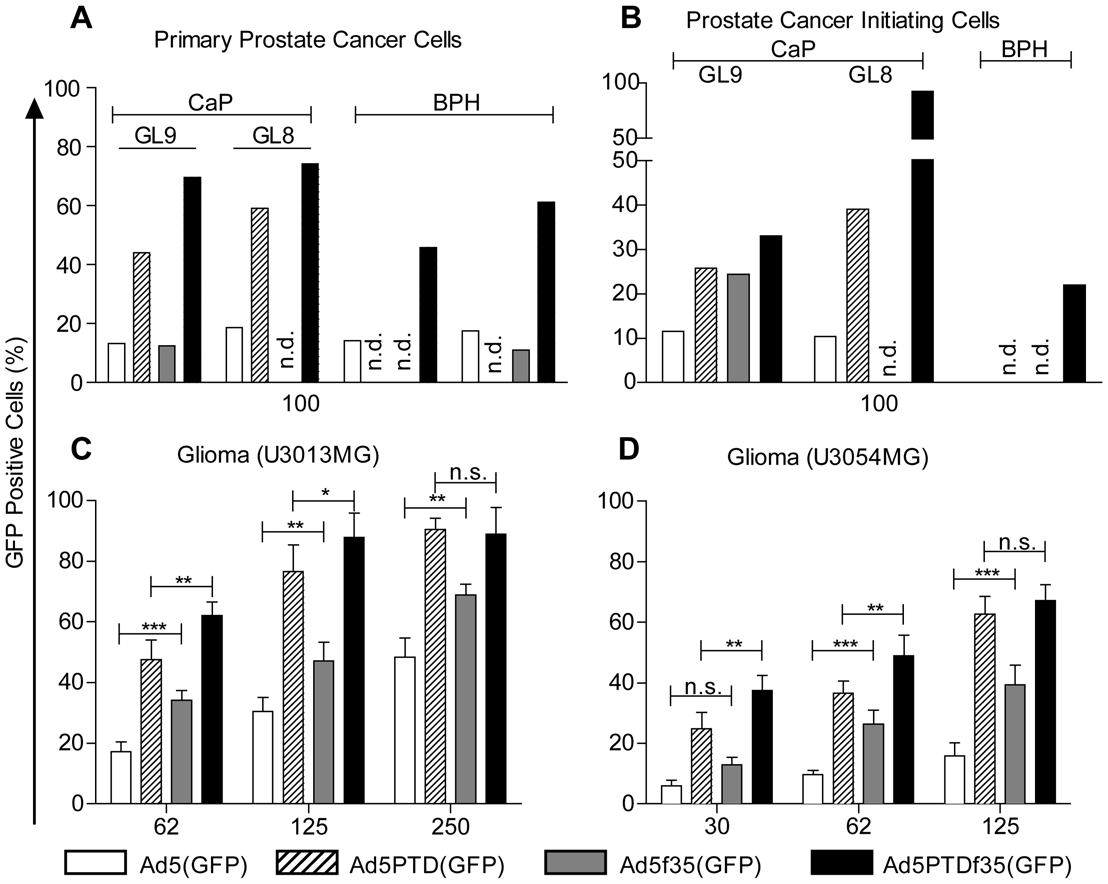
Transduction efficiency of Ad5(GFP), Ad5PTD(GFP), Ad5f35(GFP) and Ad5PTDf35(GFP) in human primary tumor cells. Primary human tumor cells were transduced with the four GFP-encoding adenoviral vectors using fixed evg/cell and fixed total volumes. GFP expression was then analyzed by flow cytometry and transduction level is presented as the percentage of GFP-positive cells. The numbers under the x-axes indicate the evg/cell used for transduction. Experiments were repeated three times for the two primary glioma cell cultures (U3013MG and U3054MG) and performed once for each individual sample for primary prostatic cells (due to low numbers of cells). CaP: prostate cancer. GL: Gleason score. BPH: benign prostatic hyperplasia. n.d.: not done. Prostate cancer initiating cells were sorted as the CD133^+^ α_v_β_1_^high^ population of primary prostate cancer cells. Error bar represents standard deviation. Non-parametric student *t*-test was used for comparison between different groups. *:p<0.05, **:p<0.01, ***:p<0.001.

Ad5(GFP), Ad5f35(GFP), Ad5PTD(GFP) and Ad5PTDf35(GFP) were evaluated side-by-side using evg/cell as indicated in Figure 2 and Figure 3 on various primary human cell types. T Cells (1×10^6^), monocytes (1×10^6^), immature DCs (1×10^5^), macrophages (2×10^4^), exocrine cells (2×10^5^), MSCs (2×10^4^), primary prostate cancer cells (2×10^5^) and primary glioma cells (2×10^5^) were transduced in suspension with appropriate amount of virus in 200 µl culture medium for 2 hours (except the primary prostate cancer cells which were transduced for 1 hour) at 37°C and thereafter the cells were washed and seeded into 24-well plates with 500 µL culture medium. Exocrine pancreatic cell clusters were allowed to adhere in 24-well plates overnight before transduction while pancreatic islets were directly transduced within 24 hours from the time of isolation. Fifty pancreatic islets with similar size were manually picked and transduced with each virus in suspension for 2 h assuming that one islet contains 1000 cells. Single cell suspensions of islets were prepared using accutase treatment before flow cytometry analysis. All samples were analyzed by flow cytometry (BD FACS Canto II, BD Biosciences, San Jose, CA) 48 hours post-transduction. Experiments were repeated on cell cultures from 4–8 different donors (heamatopoietic cells, pancreatic exocrine cells, pancreatic islets) or repeated on the same donor sample three times (glioma cells and MSCs). The data for prostate cancer cell samples are shown for each individual. Non-parametric student *t*-test was used for comparison between different groups.

### Biodistribution Study

Balb/c mice were purchased from Taconic Inc. (Ry, Denmark) and housed in individually ventilated cages (3 mice per cage). Mice were injected intravenously with Ad5(GFP), Ad5PTD(GFP), Ad5f35(GFP) and Ad5PTDf35(GFP) at a dosage of 5×10^9^ evg/mouse in 200 µL total volume. Blood, bone marrow (BM), brain, heart, kidney, liver, lung, ovary and spleen were harvested 48 hours post virus injection. Tissue samples (approximately 5 mg), blood (100 µL) or bone marrow (1×10^6^ cells) were lysed using 200 µL ATL buffer (Qiagen, Hamburg, Germany). Viral DNA was isolated from tissue lysate using High Pure Viral Nucleic Acid Kit (Roche Applied Science, Bromma, Sweden). The presence of viral genome DNA was detected by quantitative PCR with specific primer detecting the adenoviral E4 region as previously described [10].

### *Ex vivo* Expansion of Cytomegalovirus-specific T cells Using Adenovirus-transduced DCs

Monocytes from four CMV-seropositive, HLA-A2-positive blood donors were isolated from PBMCs by plastic adhesion for 90 min, as described above. After washing away the non-adherent lymphocyte fraction, the monocytes were detached and transduced with Ad5(pp65) or Ad5PTDf35(pp65) at 100 evg/cell. The Ad-transduced monocytes were then differentiated to fast DCs by growing them in culture medium supplemented with GM-CSF (50 ng/mL, Gentaur) and IL-4 (25 ng/mL, Gentaur) for 3 days with fresh medium every day. DCs were then maturated by adding TNF-α (40 ng/ml, Invitrogen) and poly(I:C) (30 µg/mL, Sigma-Aldrich, St Louis, MO) at day 3 and cultured for 24 hours as previously described [22]. The transduced and matured DCs were then mixed with autologous T cells at a 1∶10 ratio and cultured for 11 days to stimulate and expand antigen-specific T cells. The frequency of CMV-pp65-specific T cells were evaluated by HLA-A*0201/pp65_459–503_ tetramer (PE-conjugated, Beckman Coulter, San Diego, CA) staining before (pre-stim) and 11 days after stimulation (post-stim).

## Results and Discussion

### Combined Genetic Engineering of the Ad5 Hexon (Tat-PTD) and Fiber (f35) Significantly Increases the Transduction Capacity of a Variety of Primary Human Cell Types

Genetic modification of T cells, monocytes, macrophages and DCs are of great interest for immunotherapy and genetic vaccination strategies. We therefore investigated the transduction capacity of our capsid-modified vectors on different subsets of human hematopoietic cells isolated from healthy blood donors. Both non-activated and activated CD3^+^ T cells from eight donors were transduced at 2000 evg/cell. We found that activated T cells are easier to transduce with Ad5 vectors than non-activated T cells (Figures 2A and 2B). The double-modified Ad5PTDf35(GFP) vector with both hexon and fiber modification had significantly higher transduction capability (p<0.001) than the other vectors (Figures 2A and 2B), suggesting that Ad5PTDf35-based vectors could be of interest for modification of T cell genomes by zinc-finger and/or TALE nucleases [28]. Transduction of CD14^+^ monocytes, monocytes-derived immature DCs and monocytes-derived macrophages were evaluated in the same manner. The fiber 35-based vectors, *i.e.*, Ad5f35(GFP) and Ad5PTDf35(GFP), had significantly higher transduction capacity of monocytes than the fiber 5-based vectors Ad5(GFP) and Ad5PTD(GFP), (Figure 2C). For immature DCs (Figure 2D) and macrophages (Figure 2E), the double-modified Ad5PTDf35(GFP) vector was the most efficient (p<0.001). Our data suggest that Ad5PTDf35-based vectors should be evaluated in genetic vaccination strategies for optimal uptake by DCs.

The immunosuppressive properties of mesenchymal stem cells (MSCs) make them attractive in association with allogeneic cell and organ transplantation. They are also known to migrate to tumors, which make them suitable for use as carriers of oncolytic viruses to tumor sites. Human MSCs were transduced with vectors at different evg/cell ratios. Individual modifications of either the Ad5 hexon (Tat-PTD) of fiber (f35) dramatically increased the transduction capacity. However, the double-modified Ad5PTDf35(GFP) vector was the most efficient with transduction levels of up to 99% (Figure 2F). The data suggests that MSCs are potential carriers of oncolytic Ad5PTDf35-based viruses to tumor metastases.

Transplantation of pancreatic islets is today an attractive alternative to whole organ transplantation for a subset of patients with type-1 diabetes [29]. Islets can be modified with adenoviral vectors to over-express certain molecules for optimization of islet engraftment and protection from host rejection [30], [31], [32]. Genetic modification of exocrine pancreatic cells is useful to evaluate the potential to differentiate exocrine pancreatic cells into insulin-producing islet beta cells. Human pancreatic exocrine cells and islets were transduced with vectors at different evg/cell ratios. The fiber 35-based vectors yielded significantly higher levels of transduction on both cell types compared to the fiber 5-based vectors (p<0.001), (Figures 2G and 2H). In addition, Tat-PTD-modification of the Ad5 hexon improves the transduction level of exocrine cells but not islets, which is probably due to the three-dimensional structure of the pancreatic islets, leaving only the outer cell layers of the islet exposed to the viral vectors during transduction. The results suggest that Ad5PTDf35-based vectors are particularly applicable for experimental research on pancreatic cells.

### Ad5PTDf35 Shows Significantly Increased Transduction Capacity of Primary Human Tumor Cells

Oncolytic adenoviruses are attractive agents for cancer therapy and they also have the capacity to kill drug- and radiation-resistant cancer cells with stem-cell like properties, also known as tumor initiating cells or cancer stem cells [33]. We therefore investigated the vectors on primary human prostatic epithelial cells. The double-modified Ad5PTDf35(GFP) vector showed the highest transduction capacity in of both benign prostatic hyperplasia (BPH) and prostate cancer cells of Gleason (GL) score 8 and 9 (Figure 3A). Importantly, the beneficial effect of the Ad5PTDf35(GFP) vector was particularly pronounced when evaluated for transduction of the CD133^+^ α_v_β_1_^high^ population of prostate cancer initiating cells [34] where the increase was 3-fold (GL9) and 10-fold (GL8) compared to Ad5(GFP), (Figure 3B). Primary high-grade human glioma cells (glioblastoma multiforme grade IV) labeled U3013MG and U3054MG were evaluated in the same manner and we found that the Tat-PTD-modification increased the transduction capacity of both fiber 5-based and fiber-35 based adenoviral vectors (Figures 3C and 3D). Taken together, our data suggests that Ad5PTDf35-based oncolytic adenoviruses can efficiently transduce primary glioma and prostate cancer cells and thus may be of therapeutic value for targeting primary cancer cells and more importantly cancer initiating cells, which are often multidrug resistant as well as radiotherapy resistant.

### Biodistribution of Surface-modified Virus in Mice After Systemic Administration

**Figure 4:**
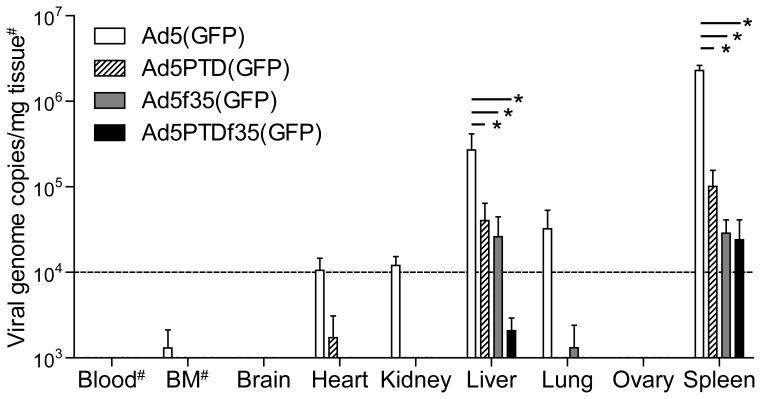
Biodistribution of surface-modified adenoviruses in mice after intravenous injection. Ad5(GFP), Ad5PTD(GFP), Ad5f35(GFP) and Ad5PTDf35(GFP) virus vectors (evg = 5×10^9^) were injected intravenously (tail-vein) into female balb/c mice and organs were harvested 48 hours post-virus injection. Viral genome DNA was isolated from the various organs and quantified by quantitative PCR in triplicates. Viral genome copy per tissue weight (mg) are shown as mean+SD (n = 3). One-way ANOVA with Tukey post test was used for comparison between different groups. #: Blood values are based on 100 µl blood from sacrificed mice and bone marrow (BM) values are based on 5×10^6^ cells. *:p<0.05.

Oncolytic adenoviruses are under evaluation as anti-cancer agents by many research groups and companies. Since the tropism of our surface-modified viruses is altered, it was of interest to investigate their biodistribution in mice after systemic administration (tail-vein injection). The wild-type virus vector, Ad5(GFP), was detected in the spleen (2.3×10^6^ copies/mg tissue), liver (2.7×10^5^ copies/mg tissue), lung (3.2×10^4^ copies/mg tissue), kidney (1.2×10^4^ copies/mg tissue) and heart (1.0×10^4^ copies/mg tissue), (Figure 4). The hexon HVR5-PTD-modified virus, Ad5PTD(GFP), was mainly detected at lower levels in the spleen (9.5×10^4^ copies/mg tissue) and liver (3.8×10^4^ copies/mg tissue). The same was true for the fiber-chimeric virus, Ad5f35(GFP), which was mainly detected at lower levels in the spleen (2.8×10^4^ copies/mg tissue) and liver (2.5×10^4^ copies/mg tissue). The double-modified virus, Ad5PTDf35(GFP), was only detected at low levels in the spleen (2.4×10^4^ copies/mg tissue) and liver (2.0×10^3^ copies/mg tissue).

Ad5 vectors are known to be sequestrated in mouse liver with potential toxicity due to hepatocyte transduction. This can hamper the therapeutic effect of systemic delivery of oncolytic adenovirus to treat metastatic cancers at other sites than the liver, at least in mouse models. Several groups have reported that the Ad5 fiber shaft and knob and/or the capsid (hexon) play an important role in liver cell transduction. The binding of the KKTK domain on the fiber shaft to heparin sulfate proteoglycans (HSPGs) has been shown to mediate liver uptake [3], [35], [36]. Blood coagulation factor IX (FIX) and complement C4-binding protein (C4BP) bridge the Ad5 fiber knob to HSPGs and low-density lipoprotein receptor-related protein (LRP) on hepatocytes [37], and coagulation factor X (FX) binds to HVRs of the hexon of the Ad5 virus capsid leading to liver transduction [38], [39], [40]. A number of mutations have been reported to completely abolish or compromise CAR-dependent transduction. However, neither a S408E mutation in the fiber knob AB loop [41], a Y477A mutation in the fiber knob DE loop [37], [42] nor deletion of TAYT in the fiber knob FG loop [43] reduced liver transduction. In contrast, the CAR binding-abolished viruses AdL.F* and dl-K420A-Z possess approximately 10-fold reduced liver transduction capacity, wherein AdL.F* contains four mutations (R412S, A415G, E416G, K417G) in the AB loop of the Ad5 fiber knob plus an insertion in the HI loop of the fiber knob [44] and dl-K420A-Z harbors a mutation (K420A) in the Bβ-sheet of the fiber knob [45].

As previously reported [46], we also detected liver sequestration of wild-type Ad5. However, by either fiber-replacement, Ad5f35(GFP), or hexon HVR5-modification, Ad5PTD(GFP), liver transduction was reduced approximately 10-fold (p<0.05). The reduction in liver transduction by the PTD-modified virus could be explained by sequence alteration in hexon HVR5 site leads a reduced binding of blood factor FX to hexon [10], [40]. The reduction in liver uptake of the fiber-replaced virus is also in accordance with other reports [44], [45], [47], [48], which could be explained by that a change in both the fiber shaft and knob leads to a reduction in of binding of HSPGs and blood factors C4BP, FIX and FX. Importantly, the double-modified virus, Ad5PTDf35, reduced liver sequestration by approximately 100 times, indicating a synergistic effect by modifying both the fiber and capsid.

High level of viral genome was present not only in the liver but in the spleen as well, suggesting that splenocytes also play a role in viral clearance and degradation. However due to that the weight of liver (ca. 1000 mg) is higher than that of spleen (ca. 80 mg), liver is still the predominant organ for adenovirus sequestration. In general, our surface-modified viruses were recovered to a lesser extent compared to the wild-type Ad5(GFP), suggesting a faster *in vivo* clearance of the surface-modified viruses.

### Ad5PTDf35(pp65)-modified DCs are Better than Ad5(pp65)-modified DCs for *ex vivo* Expansion of CMV pp65-specific T Cells

**Figure 5:**
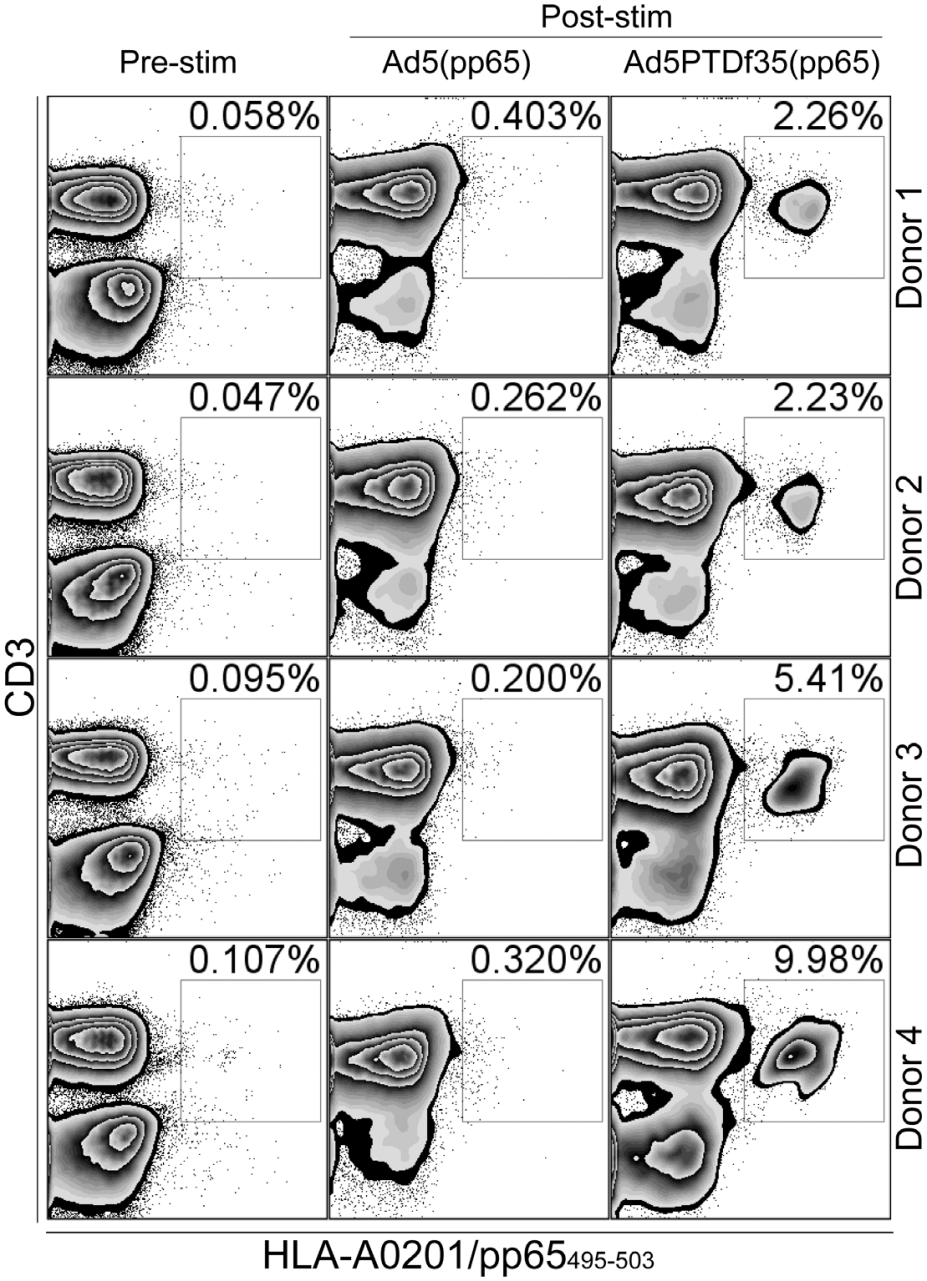
*Ex vivo* expansion of CMV pp65-reactive T cells using adenovirus-transduced DCs. DCs generated from four CMV-seropositive, HLA-A2-positive blood donors were transduced with 100 evg/cell of Ad5PTDf35(pp65) or Ad5(pp65). After expression of the pp65 protein from the viral vector inside the DC the protein is cleaved by proteasomes and the pp65_459–503_ peptide is presented by HLA-A2 on the surface of the DCs. The pp65-modified DCs were co-cultured with autologous T cells for 11 days to specifically stimulate and expand CMV pp65_459–503_-reactive CD8^+^ T cells. The frequency of CMV-pp65-specific T cells (CD3^+^) were evaluated by HLA-A*0201/pp65_459–503_ tetramer staining before (pre-stim) and 11 days after stimulation (post-stim).

To demonstrate the beneficial effect of using an Ad5PTDf35-based vector for gene delivery we chose to transduce DCs to express an antigen with the intention to use the modified cells to expand antigen-specific T cells. We have previously shown that a population of CMV pp65_495–503_-specific T cells can be significantly expanded if T cells from CMV-seropositive, HLA-A2-positive blood donors are stimulated by Ad5(pp65)-transduced autologous DCs [16], [22]. However, large amounts of viral vector need to be used to obtain efficient transduction of DCs. Since monocytes and DCs are far more efficiently transduced with the Ad5PTDf35(GFP) than with the Ad5(GFP) vector, as shown in Figure 2, we speculated that pp65_495–503_-specific T cells could be expanded even more efficiently *ex vivo* by using an Ad5PTDf35-based vector to modify the stimulator DC cells. We therefore constructed a non-modified Ad5(pp65) and a double-modified Ad5PTDf35(pp65), which both express the full-length CMV pp65 transgene. We transduced monocytes with Ad5(pp65) or Ad5PTDf35(pp65) at a relatively low dosage (100 evg/cell), differentiated them into DCs [16], [22] and used them to stimulate and expand autologous pp65-specific T cells. The Ad5PTDf35(pp65)-transduced DC stimulation increased the pp65_495–503_-reactive T cells population for all donors, approximately 50–100 fold, while Ad5(pp65)-transduced DC stimulation only increased the pp65_495–503_-reactive T cell population 2–8 fold (Figure 5). These data clearly show that Ad5PTDf35(pp65) would be highly efficient for DC modification to expand CMV pp65-specific T cells *ex vivo*. Expanded donor-derived T cells could then be adoptively transferred to transplant patients experiencing CMV complications due to their immune suppressive medication [49]. Furthermore, this example serves as an indication that Ad5PTDf35-based vectors are superior to Ad5-based vectors in other settings as well.

### Construction of AdEasy System-compatible Backbone Plasmids Containing Hexon (Tat-PTD) and/or Fiber (f35) Modifications for Recombinant Ad5 Vector/Virus Production

In order to make our constructs readily available as research tools we adapted the modifications into plasmids compatible with the AdEasy system [17], [19], where recombinant adenovirus DNA is constructed in homologous recombination-proficient *Escherichia coli* bacteria (stain BJ5183). The technology is based on two plasmids, pShuttle (cloning plasmid for the transgene of choice) and pAdEasy (virus backbone plasmid). The pShuttle plasmid allows one to easily clone the gene of interest in the adenoviral E1 region. The entire recombinant adenovirus genome with the gene of interest is then constructed by homologous recombination between the pShuttle(trangene) plasmid and the pAdEasy(backbone) plasmid. Adenovirus particles are produced by transfecting the linearized double-stranded DNA virus genome into producer cells such as HEK-293T or 911. Since this is a relatively easy technology to produce recombinant adenoviruses, it is today a widely used system for recombinant adenovirus construction. The standard AdEasy backbone plasmid that is available today will generate Ad5 vectors without any surface-modification. Due to the beneficial effect observed with our surface-modified Ad5 vectors we decided to clone the sequences leading to these modifications into various pAdEasy backbone plasmids to generate user-friendly AdEasy-compatible backbone plasmids with hexon (Tat-PTD) and/or fiber (f35) modifications. All the adenovirus backbone plasmids are listed in Table 1. We believe that they will serve as useful tools to advance the development of genetic vaccine vectors, gene delivery vectors and oncolytic adenoviruses.
